# How Do We Explore Heterogeneity in Turnover of Musculoskeletal Proteins?

**DOI:** 10.1093/function/zqab034

**Published:** 2021-06-28

**Authors:** Jakob Agergaard, Michael Kjær

**Affiliations:** Institute of Sports Medicine Copenhagen, Department of Orthopedic Surgery, Copenhagen University Hospital—Bispebjerg and Frederiksberg, 2400 Copenhagen NV, Denmark; Center for Healthy Aging, Department of Clinical Medicine, University of Copenhagen, 2200 København N, Denmark; Institute of Sports Medicine Copenhagen, Department of Orthopedic Surgery, Copenhagen University Hospital—Bispebjerg and Frederiksberg, 2400 Copenhagen NV, Denmark; Center for Healthy Aging, Department of Clinical Medicine, University of Copenhagen, 2200 København N, Denmark

## A Perspective on “A novel stable isotope approach demonstrates surprising degree of age-related decline in skeletal muscle collagen proteostasis”

Stable isotope amino acid tracer methodology has for many years been the backbone in determining the protein turnover rate of the whole body as well as tissue specific proteins.^1^ Within the area of musculoskeletal research, infusion of amino acid tracers has been widely used to study the anabolic and catabolic effects of interventions such as exercise, inactivity/immobilization, aging, nutrition, or pharmacological treatments. In the early days, radiolabeled tracers were used, but now gradually replaced by nonradioactive stable isotopes attached to a variety of amino acids. Yet, such measurements with amino acid tracer infusions are limited to hours under very constrained settings. In the recent years, an increasing focus has been drawn to the application of deuterium as an isotope tracer with oral intake of D_2_O as the route of administration. The use of deuterium as an isotope tracer dates back to the 1930s, where deuterium was used to measure intermediary metabolism. Advances within mass spectrometry have now made it possible to determine even low levels of deuterium enrichment. Therefore, over the past decade, a growing number of studies have utilized oral intake of D_2_O with the subsequent deuterium labeling of alanine as a precursor for measuring either protein synthesis or protein breakdown over following days or weeks. This approach has expanded the field, enabling integrated measurements of protein turnover rates during anabolic and catabolic situations, which are not limited by the same constraints as studies with infusion of a stable isotope amino acid tracer.

Whether measuring the protein turnover rate over hours with stable isotope amino acid tracers or with deuterium labeling over days or weeks, the following mass spectrometry to determine the product/protein enrichment of the tracer is in general performed on whole tissue homogenates or larger tissue fractions. As an example, Miller and colleagues in a single study explored the exercise effect on muscle sarcoplasmic, myofibrillar, and collagen protein synthesis as well as tendon collagen synthesis simultaneously.^2^ Although this methodological approach is still widely used and has provided valuable information for the current knowledge base of musculoskeletal protein turnover, the resolution on a potential heterogeneity within the tissue cannot be mapped. Therefore, to expand on the solid knowledge base, the strive for the future research would be to elucidate if a greater complexity in protein turnover is seen if focus is put on single proteins, specific regions, or pools of proteins within the tissue.

In the present issue of *Functions*, Abbott *et al* conducted a study on young and old rats with 60 d of D_2_O administration in order to explore the aging effect on myofibrillar and intramuscular collagen turnover.^3^ They observed that in the muscle collagen protein synthesis is much lower than myofibrillar protein synthesis, which has previously been shown in several studies. However, they also demonstrate that due to the low turnover of the intramuscular collagen protein, only approximately 20% of the collagen pool is dynamic. Furthermore, this dynamic pool seems to be lower in old compared to young rats. This indicates a loss of proteostatic maintenance with aging, which could be associated with an increased intramuscular collagen content, and thereby connective tissue disposition, which is seen within the muscle of the old rats.

Within the area of tendon and connective tissue research, the approach of distinguishing between dynamic and inactive or slow and fast pools of proteins is an interesting approach. We have previously demonstrated with carbon-14 measurements on human Achilles tendons that the core of the tendon hardly renews after puberty, indicating that tendons do have a pool of proteins with an inactive or very slow turnover rate,^4^ and a similar low protein turnover has also been found in cartilage.^5^ By proteomics applied on horse Achilles tendons, heterogeneity in the protein composition between the fascicular and the interfascicular matrices has been shown,^6^ and in a recent study, the same group showed on rat Achilles tendons that the protein category with the lowest turnover rates was the collagen, which is highly abundant in the fascicular matrix.^7^ Furthermore, they demonstrated that the same protein, collagen type I, had a higher turnover rate when located in the interfascicular matrix than when located in the fascicular matrix. Such tissue heterogeneity must be taken into account when exploring which effects exercise, aging, or pharmacological interventions pose on healthy as well as diseased tendons. In other words, a high tissue resolution is needed in order to discriminate which pools or regions are affected.

Recently, Camera and colleagues performed a study with deuterium labeling through administration of D_2_O during a resistance training study and subsequent dynamic protein profiling (DPP) on muscle biopsies.^8^ Through the DPP, deuterium-labeled and label-free peptides were measured in order to determine the synthesis and breakdown rates, as well as the abundance of single proteins from the muscle homogenate in response to the resistance exercise. Advances within secondary ion mass spectrometry (SIMS), as either time-of-flight SIMS or NanoSIMS, will also be of great interest in the future to study tissue heterogeneity.^9^ With this approach, *in situ* detection of tracer labeling from tissue sections will elucidate possible region-specific differences in protein turnover. Therefore, DPP and SIMS are important approaches to be considered for the future toolbox of methods to determine protein turnover rates of musculoskeletal tissues, to be able to distinguish between dynamic and inactive protein pools, or between pools with a slow or fast protein turnover rates. Hopefully any method that goes beyond the whole tissue or larger tissue fraction approach will help put a greater resolution on the complexity of protein turnover and thereby paves the road for the future research in musculoskeletal tissue, whether it is bone, cartilage, tendon, ligament, or skeletal muscle.

## Funding

None declared.
